# Effect of *Nelumbo nucifera* Petal Extracts on Lipase, Adipogenesis, Adipolysis, and Central Receptors of Obesity

**DOI:** 10.1155/2013/145925

**Published:** 2013-11-14

**Authors:** Chandrasekaran Chinampudur Velusami, Amit Agarwal, Vijayalakshmi Mookambeswaran

**Affiliations:** ^1^R&D Centre, Natural Remedies Pvt. Ltd., Plot No. 5B, Veerasandra Industrial Area, 19th K.M. Stone, Hosur Road, Bangalore, Karnataka 560100, India; ^2^Center for Bioseparation Technology, VIT University, Vellore, Tamil Nadu 632014, India

## Abstract

*N. nucifera* is one among the important medicinal plants assessed for its antiobesity action in various preclinical models. The present study was aimed at investigating the antiobesity effect of methanol and successive water extracts of petals of *N. nucifera* by studying its effect on adipogenesis, adipolysis, lipase, serotonin (5-HT_2C_), cannabinoid (CNR_2_), melanocyte concentrating hormone (MCHR_1_), and melanocortin (MC_4_R) receptors. Both methanol and successive water extracts of *N. nucifera* petals had an effect on inhibition of lipid storage in adipocytes and on increasing lipolysis. *N. nucifera* petal methanol extract exhibited the concentration-dependent inhibitory effect on lipase activity with an IC_50_ value of 47 *µ*g/mL. *N. nucifera* petal extracts showed evident agonist and antagonist activity towards 5-HT_2C_ and CNR_2_ receptors, respectively, while it showed no effect towards MCHR_1_ and MC_4_R receptors. Overall, methanol extract of *N. nucifera* petals showed better activity than successive water extract.

## 1. Introduction

Several herbs have been indicated for weight management [1]. One such plant used for weight management is *Nelumbo nucifera *Gaertn*. N. nucifera*, known by a number of names including Indian lotus, sacred lotus, bean of India, or simply lotus, is one of two species of aquatic plant in the family Nelumbonaceae. Almost all parts of *N. nucifera* are edible, and in many Asian countries it was found in the recipe of food [2]. Extracts of *N. nucifera *flowers, seeds, rhizomes, and leaves have been reported to have varied therapeutic potential including antistress [3], antiobesity [4], antioxidant [5], hepatoprotective [6], antidiabetic activity [7], anti-inflammatory [8, 9], antipyretic [10], antibacterial [11], and immunomodulatory [12, 13] activities.

Several bioactive phytocompounds derived from these plant parts were belonging to different chemical groups, including alkaloids, flavonoids, glycosides, triterpenoid, and vitamins [14]. Leaves, root, and the embryonic stage of *N. nucifera* have been reported to contain alkaloids such as roemerine, nuciferine, nornuciferine, nelumboside, anonaine, 5-methoxy-6-hydroxyaporphine, liensinine, and asimilobine [15]. Bisbenzylisoquinoline alkaloids from *N. nucifera *were shown to be bioavailable after oral administration to rats at a dose of 20 mg/kg [16]. *N. nucifera *alkaloid was shown to inhibit 3T3-L1 preadipocyte differentiation and improve high-fat diet-induced obesity and body fat accumulation in rats [17].

Several flavonoids and nonflavonoids from flowers of *N. nucifera* reported by several authors were consolidated in a review by Mukherjee et al. [14]. Flavonoids include myricetin-3-O-b-D-glucopyranoside, quercetin-3-O-b-D-glucuronide, astragalin, quercetin, 3,4-dihydroxybenzoic, kaempferol, p-hydroxybenzoic acid, and b-sitosterol which were isolated from ethanol extract of the petals of *N. nucifera* [18]. Nonflavonoid compounds, including adenine, myo-inositol, arbutin, and sitosterol glucopyranoside, were identified in flower extract [14]. Wu et al. [19] demonstrated the antiobesity effect of a flavonoid-enriched extract from *N. nucifera* leaf (NLFE) in high-fat diet (HFD) fed C57BL/6 mice and concluded its action via lipid-regulated enzymes, thereby attenuating body lipid accumulation and preventing obesity. Antiobesity action of leaves and seeds of *N. nucifera* was extensively studied in *in vitro* and *in vivo* models by many researchers [4, 20–22]. The present study was designed to investigate the effect of *N. nucifera *petal extracts on lipase, adipogenesis, adipolysis, and central receptors *in vitro*.

## 2. Materials and Methods

### 2.1. Chemicals

Dexamethasone, isobutylmethylxanthine (IBMX), oil red O, porcine lipase enzyme, thiazolyl blue tetrazolium bromide (MTT), 4-methyl umbelliferyl oleate, GW 803430, melanocyte concentrating hormone (MCH), melanotan II, DL-isoproterenol hydrochloride, and orlistat were procured from Sigma-Aldrich. AM630, CP 55,940, AL34662, cell plating reagent-3, cell assay buffer, substrate reagent 1, and substrate reagent 2 were obtained from DiscoveRx (USA). Insulin was procured from Biocon. Fetal bovine serum (FBS) and bovine calf serum (BCS) were purchased from Hyclone. Enzychrom glycerol detection kit was procured from BioAssay systems. DMEM was obtained from Gibco Life Technologies.

### 2.2. Plant Material

The petals of *N. nucifera* (100 g each) were procured from a local commercial supplier and were authenticated at National Institute of Science Communication and Information Resources (NISCAIR), New Delhi. A voucher specimen (no. 811) was deposited in our herbarium. Dried petals were extracted with methanol (~400 mL) by refluxing at 70°C for 1 hour. Extract solution was filtered, and the remaining raw material was subjected to methanol extraction by repeating the above steps twice. The liquid filtrates were combined and concentrated by distillation under vacuum to a thick paste, followed by drying under vacuum at temperature 70°C. The dried extract was named as methanol extract and utilized to perform *in vitro* experiments. Phytochemical investigation of methanol extract of *N. nucifera* was carried out by subjecting methanol extract to HPLC analysis to identify the flavonoids as per the method described by Xingfeng et al. [23].

Methanol extraction of the raw material was carried out as mentioned above. The leftover raw material after methanol extraction was further boiled with water at 85–90°C (3 times each with 500–600 mL water for 1 h) and filtered each time. The liquid filtrates were combined and concentrated by distillation under vacuum to a thick paste, followed by drying under vacuum at temperature 80°C. The dried extract was named as successive water extract and used to perform *in vitro* experiments.

### 2.3. Cell Lines and Culture Conditions

3T3-L1 cell line was procured from American Type Culture Collection (ATCC). 3T3-L1 fibroblasts were cultured in DMEM supplemented with 10% BCS and incubated at 37°C; 5% CO_2_. The U2OS cell line coupled with 5-HT_2C_ or MC_4_ receptor and CHO-K1 cell line coupled with CNR_2_ or MCHR_1 _receptor were obtained from DiscoveRx. U2OS and CHO-K1 cells were maintained in cell plate reagent in 96-well tissue culture plates for 48 h and 24 h, respectively.

### 2.4. Cell Viability Determination

Initial experiments using 3T3-L1, CHO-K1, and U2OS cells were conducted to assess the cytotoxic concentrations of both methanol and successive water extracts of *N. nucifera*. Cell viability was determined by a colorimetric MTT assay as described by Mosmann [24]. In brief, cells were cultured in 96-well plates at a seeding density of 5 × 10^3^ cells/well. After 24 h of seeding, the cells were treated with and without *N. nucifera* extracts up to a concentration of 100 *μ*g/mL. Thereafter, the cells were rinsed and further incubated with MTT for 1 h. After 1 h, MTT crystals were dissolved in 200 *μ*L DMSO. Optical density was read at 570 nm, and, consequently, the noncytotoxic concentrations were chosen for conducting *in vitro* studies.

### 2.5. Adipogenesis Assay

Effect of *N. nucifera* petal extracts on adipogenesis was evaluated by examining their ability to inhibit the differentiation of preadipocytes to adipocytes using 3T3-L1 cells as a test system. On day 0, mouse 3T3-L1 fibroblasts were seeded at a density of 3 × 10^4^ cells/well in a 48-well plate containing DMEM supplemented with 10% BCS. On day 1, cells were changed to DMEM medium supplemented with FBS (5%), IBMX (0.5 mM), insulin (10 *μ*g/mL), and dexamethasone (1 *μ*M) with and without *N. nucifera* extracts. Guggulsterone was used as a reference control. On day 3, cells were changed to DMEM supplemented with FBS (5%) and insulin (5 *μ*g/mL) with and without *N. nucifera* petal extracts. On days 5 and 7, the cells were changed to DMEM supplemented with 5% FBS. On day 8, 3T3-L1 adipocytes were rinsed with PBS and fixed using 10% formalin for 30 min followed by another rinse with 60% isopropanol solution and allowed to dry. The cells were stained with oil red O solution (0.5% oil red O in isopropanol, diluted in proportion of 3 parts of oil red O stock and two parts of distilled water) for 15 min at room temperature. Dye retained in adipocytes was extracted with isopropanol and quantified by measuring the absorbance at 500 nm.

### 2.6. Adipolysis Assay

Adipolysis assay was performed to evaluate the possible lipolytic activity of *N. nucifera* petal extracts by examining their ability to release glycerol from differentiated 3T3-L1 cells. On day 0, 3T3-L1 cells were seeded at a density of 3 × 10^4^ cells/well in a 48-well plate containing DMEM medium supplemented with 10% BCS. On day 1, cells were changed to DMEM supplemented with 5% FBS, IBMX (0.5 mM), insulin (10 *μ*g/mL), and dexamethasone (1 *μ*M). On day 2, cells were changed to DMEM, supplemented with FBS (5%) and insulin (5 *μ*g/mL), and left undisturbed for 2 days. On day 4, the cells were allowed to grow in FBS (5%) for consecutive three days. On day 7, the cells were starved overnight with DMEM containing 2% BSA. On day 8, cells were treated with noncytotoxic concentrations of *N. nucifera* extracts and DL-isoproterenol hydrochloride (reference control) separately for 4 hr in KRB (Krebs-Ringer Bicarbonate) buffer (pH 7.2). The supernatant was collected and estimated for glycerol content by adding 100 *μ*L of glycerol working reagent and 10 *μ*L of sample/standard per well in a 96-well assay plate. Plate was tapped to mix and incubated at room temperature for 20 min. Read the color intensity at 570 nm in VersaMax plate reader.

### 2.7. Lipase Assay

The inhibitory effect of *N. nucifera* on porcine pancreatic lipase was evaluated. The assay was based on the principle of conversion of the substrate 4-methyl umbelliferyl oleate to 4-methyl umbelliferone by an active porcine lipase enzyme [25]. In brief, the total reaction volume of 50 *μ*L contained 15 *μ*L of Tris buffer/reference control (orlistat)/*N. nucifera* extract, 5 *μ*L of lipase enzyme, 5 *μ*L of demineralized water, and 25 *μ*L of substrate (4-methyl umbelliferyl oleate). Mix these reagents and determine the change in fluorescence at 25°C for 20 min at an excitation and emission wavelength of 360 nm and 460 nm, respectively, using FLUOstar Optima.

### 2.8. Receptor Assays

Both methanol and successive water extracts of petals of *N. nucifera *were screened for possible agonistic and antagonistic activity towards selected receptors at a concentration of 10 *μ*g/mL.

#### 2.8.1. Agonist Assays

5*HT*_2*C*_ *and MC*_*4*_ *R Receptor Assays*. The U2OS cell line coupled with either 5-HT_2C_ or MC_4_R receptor was plated at a density of 10^4^ cells/well in 96-well tissue culture plates, containing cell plating reagent. After 48 h incubation, *N. nucifera* petal extracts or reference agonist, (AL34662 for 5-HT_2C_; melanotan II for MC_4_R) were added in separate wells at noncytotoxic concentrations and incubated for 90 min at 37°C; 5% CO_2_. 55 *μ*L of prepared detection reagent solution was added to each well. After 60 min incubation at room temperature, the plate was read using luminescence plate reader (FLUOstar).

#### 2.8.2. Antagonist Assays

*Cannabinoid Receptor 2 (CNR*_*2*_ *).* Effect of both methanol and successive water extracts of *N. nucifera* on inhibition of CP 55,940 (CNR_2_ agonist) elicited CNR_2_ activity in Gi/Go coupled CHO-K1 cell line was studied. 10^4^ cells/well were plated in 96-well tissue culture plates containing cell plating reagent. After 48 h incubation, *N. nucifera* or reference antagonist (AM630) was added to the respective wells and incubated for 30 min at 37°C; 5% CO_2_. 5 *μ*L of agonist compound (CP 55,940) was added to the respective wells in a final volume of 110 *μ*L and incubated for 90 min. 55 *μ*L of prepared detection reagent solution was added to each well and incubated for 60 min at room temperature, and plate was read using luminescence plate reader (FLUOstar).

*Melanin Concentrating Hormone Receptor (MCHR*_*1*_ *) Assay.* Antagonistic potential of *N. nucifera *extracts on MCHR_1_ receptor was studied using MCHR_1_ Gi coupled CHO-K1 cells. Cell density of 3 × 10^4^ cells/well was plated in 96-well tissue culture plates containing cell plating reagent and incubated for 24 h. After incubation, the entire medium was aspirated and 45 *μ*L of cell assay buffer and antibody mixture was added to each well. *N. nucifera* or reference antagonist (GW 803430) was added to the respective wells and incubated for 15 min at 37°C and 5% CO_2_. Agonist compound (MCH—62.5 nM + Forskolin 20 *μ*M) was added to the respective wells and incubated for 30 min. 60 *μ*L of prepared detection reagent solution and cAMP solution D was added to each well and incubated for 60 min at room temperature in the dark. 60 *μ*L of cAMP Solution A was added and incubated for 3 hr at room temperature in the dark. Plate was read using luminescence plate reader (FLUOstar).

### 2.9. Data Analysis

For adipogenesis and adipolysis assays, statistical analysis was performed by one-way analysis of variance using the Graphpad Prism statistical software. Results are represented as Mean ± SD from three replicates per treatment group. Differences with *P* < 0.05 in comparison to control were considered to be statistically significant. For lipase assay, mean of the relative fluorescence unit (RFU) of *N. nucifera*/reference control tested in triplicate was calculated. From the mean values, percentage inhibition (%*I*) was calculated using the following equation:

(1) $$\begin{array}{c} \%I=\frac{\left(\text{RFU}\;\text{of}\;\text{Control}-\text{RFU}\;\text{of}\;\text{Sample}\right)}{\left(\text{RFU}\;\text{of}\;\text{Control}\right)}\times 100. \end{array}$$

IC_50 _ was calculated by the Finney software. For receptor assays, Student's *t*-test was performed using GraphPad Prism 5 statistical software to test for differences among all treatments. Differences with *P* < 0.05 were considered to be significant.

## 3. Results

### 3.1. Effect of *N. nucifera* on Differentiation of 3T3-L1 Cells

**Figure 1:**
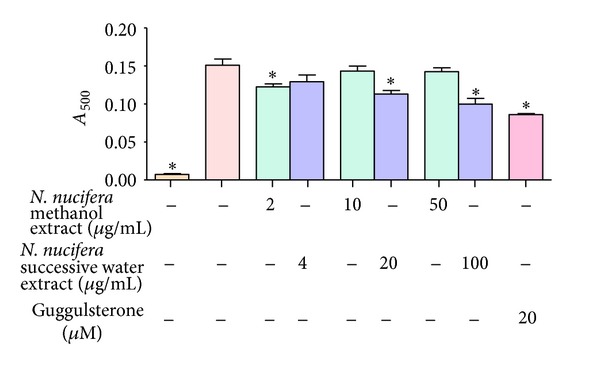
Effect of *N. nucifera* on adipogenesis using 3T3-L1 cells.

Noncytotoxic concentrations up to 50 *μ*g/mL and 100 *μ*g/mL were selected for methanol and successive water extract of *N. nucifera,* respectively, for *in vitro* cell-based assays. To test whether *N. nucifera* extracts inhibit adipocyte differentiation, the differentiated adipocytes were stained by oil red O. The staining results showed that incubation of *N. nucifera* during the differentiation period significantly inhibited 3T3-L1 adipogenesis. It was found that treatment of 3T3-L1 cells with *N. nucifera* successive water extract significantly decreased the cell differentiation and lipid accumulation in a dose-dependent manner, compared with control cells. Methanol extract of *N. nucifera *exhibited significant inhibition of adipocyte differentiation by 19% at a concentration of 2 *μ*g/mL. The reference control, guggulsterone, demonstrated a potent inhibitory activity towards lipid accumulation at 20 *μ*M with a percentage inhibition of 48 (Figure 1).

### 3.2. Lipolytic Effect of *N. nucifera* on Differentiated 3T3-L1 Cells

**Figure 2:**
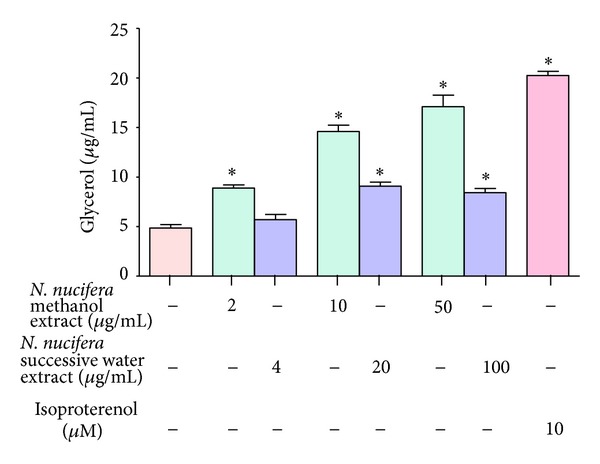
Effect of *N. nucifera* on glycerol release from differentiated 3T3-L1 cells.

*N. nucifera* petal extracts displayed lipolytic activity as evident by significant increase of glycerol release from the differentiated 3T3-L1 cells. Methanol extract of *N. nucifera* showed significant dose-dependent release of glycerol at concentrations ranging from 2 *μ*g/mL to 50 *μ*g/mL. Maximum lipolytic activity of 3.5-fold increase was observed with methanol extract of *N. nucifera* at a concentration of 50 *μ*g/mL. Successive water extract showed significant increase of glycerol levels at highest concentrations (20 and 100 *μ*g/mL), where a fold increase of 1.9 was observed. Isoproterenol, a known lipolytic agent, elicited a marked glycerol release by fat cells at the tested concentration of 10 *μ*M with a 4-fold increase over control (Figure 2).

### 3.3. Lipase Inhibitory Effect of *N. nucifera*

Pancreatic lipase inhibition of both methanol and successive water extracts of *N. nucifera* was determined at a screening concentration of 50 *μ*g/mL. Percent lipase inhibitory effect of methanol and successive water extract at 50 *μ*g/mL of *N. nucifera* was found to be 52 and 10, respectively. Further, lipase inhibitory potential of methanol extract was tested to determine the IC_50_ (the concentration required to inhibit a lipase activity by 50%). Methanol extract of *N. nucifera* exhibited a dose-dependent lipase inhibitory effect with an IC_50_ value of 47 *μ*g/mL. However, it was not more effective than orlistat which showed an IC_50_ value of 26 ng/mL (Table 1).

### 3.4. Central Target Action of *N. nucifera*

#### 3.4.1. 5-HT_**2c**_ and MC_**4**_ Receptor Assays

Both methanol and successive water extracts of *N. nucifera* at 10 *μ*g/mL showed significant equipotent stimulatory activity of about 4-fold increase towards 5-HT_2C_  receptor (Table 2). The reference control AL34662 demonstrated a potent dose-dependent agonist activity towards 5-HT_2C_ receptor with an EC_50_ value of ~10.8 nM. Both methanol and successive water extracts did not show significant agonist activity towards MC_4_ receptor at a concentration of 10 *μ*g/mL (Table 2). Melanotan II, a known MC_4_R agonist, demonstrated a concentration-dependent potent agonist activity towards MC_4_R receptor with an EC_50_ value of ~3.3 nM.

#### 3.4.2. CNR_**2**_ and MCHR_**1**_ Receptor Assays

At a concentration of 10 *μ*g/mL, methanol extract of* N. nucifera *petals displayed 26.2% antagonism against CP 55,940 activity towards CNR_2_ receptor, whereas the successive water extract was found to be inactive at a concentration of 10 *μ*g/mL (Table 2). AM630, a known CNR_2_ antagonist, demonstrated a potent antagonist activity towards CP 55,940 activated CNR_2_ receptor with an IC_50_ value of ~62.3 nM. Both of these two extracts did not show antagonist activity towards MCHR_1_ (Table 2). GW 803430, a known MCHR_1_ antagonist, demonstrated a potent antagonist activity towards MCH induced MCHR_1_ receptor with an IC_50_ value of ~13 nM.

### 3.5. Phytochemical Analysis of Methanol Extract of *N. nucifera*

HPLC analysis of crude methanol extract of *N. nucifera* confirmed the presence of flavonoids as major phytochemicals. HPLC followed by UV spectral analysis confirmed the identity of flavonoid glycosides (quercetin and kaempferol glycosides) which is in compliance with the earlier report by Xingfeng et al. [23].

## 4. Discussion

Dietary fat is not directly absorbed by the intestine unless the fat has been subjected to the action of pancreatic lipase. Therefore, pancreatic lipase is one of the most widely studied mechanisms for determining natural products and potential efficacy as antiobesity agents [26]. In this study, we report the inhibitory effects of *Nelumbo nucifera* petal extracts on pancreatic lipase. Methanol extract elicited an inhibitory effect on lipase enzyme with an IC_50_ value of 47 *μ*g/mL. Similar to our study but on lotus leaf, authors have showed antiobesity activity through a concentration-dependent inhibition of lipase enzyme and also upregulated the lipid metabolism [4]. *Nelumbo nucifera* is known as sacred lotus and found to have various pharmacologically active substances including alkaloids, flavonoids, triterpenoids, polyphenols, steroids, and glycosides [23]. A phytochemical investigation of *N. nucifera* leaves led to the isolation of eight alkaloids and some of these significantly inhibited pancreatic lipases [20]. Total flavonoids from *N. nucifera* leaves showed high inhibitory activity against porcine pancreatic lipase, *α*-amylase, and *α*-glucosidase. Also, it lowered total cholesterol, triglycerides, low-density lipoprotein cholesterol, and malondialdehyde and raised the high-density lipoprotein cholesterol *in vivo* system. Moreover, it alleviated high-fat diet-induced lipid accumulation in the liver [27].

Adipocytes primarily store triglycerides and release them in the form of free fatty acid with the change of energy demand in the body. *N. nucifera* petal extracts demonstrated significant dose-dependent inhibitory effects on lipid accumulation in 3T3-L1 adipocytes. Alkaloids isolated from *N. nucifera* showed stronger inhibitory effect on adipocyte differentiation [20] and could probably contribute to the activity of petals of* N. nucifera*. Similar to this study but on lotus seed, epicarp extracts of *N. nucifera* were studied using an *in vitro* 3T3-L1 preadipocyte cell model. Results showed that the lotus seed epicarp extracts inhibited preadipocyte differentiation to adipocyte in a concentration-dependent manner [21]. Antiobesity effect of *N. nucifera* leaves extract (NNE) using high-fat diet-induced obesity in mice was studied. NNE significantly decreased the high-fat diet-induced weight gain, parametrial adipose tissue weight, and liver triacylglycerol levels in mice. Authors concluded that NNE impaired digestion, inhibited absorption of lipids and carbohydrates, accelerated lipid metabolism, and upregulated energy expenditure [4]. 

In this study, we have demonstrated that the petal extracts of *N. nucifera* clearly exhibit lipolytic activity in a dose-dependent manner in murine 3T3-L1 fibroblasts. Previous studies have reported that a liquid leaf extract of *Nelumbo nucifera* stimulated lipolysis activity in differentiated adipocytes. Also, authors have showed that treatment of adipocyte cultures with 0.5% lotus leaf extract solution significantly increased the content of free glycerol. Likewise, cultivation of cells with 1% lotus leaf extract solution induced a significant release of free glycerol compared to control cells [28]. In another study, 50% ethanol (EtOH) extract prepared from the leaves of *N. nucifera* stimulated lipolysis in the white adipose tissue (WAT) of mice and possible involvement of beta-adrenergic receptor (beta-AR) pathway was attributed to this effect. *N. nucifera* in preventing diet-induced obesity emerged to be due to various flavonoids and that the activation of beta-AR pathway was involved, at least in part [29].

Based on the 5-HT_2C_ receptor study, *N. nucifera* petal extracts showed significant agonist activity towards 5-HT_2C_ receptor and antagonistic activity towards CNR_2_ indicating its role in central targets of obesity as appetite suppressant. Alkyl 4-hydroxybenzoates were isolated from seeds of *N. nucifera* and shown to enhance and inhibit 5-HT-stimulated inward current (I(5-HT)) mediated by the human 5-HT(3)A receptors expressed in Xenopus oocytes [30]. Similar to this study, Oh et al. [31] demonstrated that chronic treatment with ethanol extract from *Morus alba* leaves exerts an antiobesity effect in diet-induced obese mice via its direct MCH_1_ receptor antagonism. Various phytocompounds, namely, alkaloids, anthocyanin, and nonanthocyanin flavonoids, have been isolated from petals of *N. nucifera* [32, 33] and shown to have other biological activities; however, their role in antiobesity activity needs to be determined. 

Flavonoid-enriched *N. nucifera* leaf extract significantly inhibited the high-fat diet-induced abnormal blood lipids and liver damage [27]. Galleano et al. [34] consolidated the proof linking flavonoid intake with metabolic disorders, namely, obesity, hypertriglyceridemia, hypercholesterolemia, hypertension, and insulin resistance. However, a number of molecular mechanisms have been identified; the effects of flavonoids on endpoints of metabolic syndrome are still inconclusive. These convolutions were explained by the complex associations among the risk factors of metabolic syndrome, the multiple biological targets controlling these risk factors, and the high number of flavonoids (including their metabolites) present in the diet and potentially responsible for the *in vivo* effects. As a result, extensive basic and clinical research is warranted to assess the relevance of flavonoids for the treatment of metabolic syndrome [34]. 

Acute and subchronic oral toxicity studies of *N. nucifera* stamens extract in rats were performed and found to have safety threshold for acute toxicity which is above 5000 mg/kg bodyweight, and no-observed-adverse-effect level (NOAEL) of the extract for both male and female rats is considered to be 200 mg/kg/day [35]. 

In conclusion, *N. nucifera* petal extract showed antilipase activity, lipolytic and antiadipogenesis effect in adipocytes *in vitro*. *N. nucifera* extract showed agonist and antagonistic effect towards central receptors involved in food intake. Thus, it is worthwhile to further investigate *N. nucifera* petal extract and phytoconstituents for its potential pharmacological effect in metabolic disorders, in particular obesity.

## Figures and Tables

**Table 1:** Effect of *N.  nucifera* extracts on pancreatic lipase activity.

Test sample	Concentration	% Lipase inhibition	IC_50_
*Nelumbo nucifera* methanol extract	12.5 *μ*g/mL	14.78	47 *μ*g/mL
	25 *μ*g/mL	29.28	
	50 *μ*g/mL	51.81	
	100 *μ*g/mL	71.94	
	200 *μ*g/mL	89.87	
*Nelumbo nucifera* successive water extract	50 *μ*g/mL	10	—
Orlistat (reference standard)	5 ng/mL	28.31	26 ng/mL
	10 ng/mL	36.89	
	25 ng/mL	44.88	
	50 ng/mL	66.15	
	100 ng/mL	68.22	
	200 ng/mL	71.91	

**Table 2:** Effect of petal extracts of *N.  nucifera* on central receptors.

Name of the test item	Solvent	Concentration	RLU (Mean ± SD)			
			5HT_2C_	MC_4_R	MCHR_1_	CNR_2_
Negative	—	NA	35947.0 ± 410.12	2322.3 ± 111.11	NA	NA
DMSO	—	0.1%	36188.5 ± 3324.11	2405.3 ± 246.41	NA	NA
*N. nucifera *methanol extract	DMSO	10 *μ*g/mL	146121.5 ± 3987.37*	2276.5 ± 289.21 (NS)	321.5 ± 38.89 (NS)	106480.0 ± 2791.66*
*N. nucifera *successive water extract	DMEM	10 *μ*g/mL	150760.5 ± 15262.89*	2071.5 ± 282.14 (NS)	320.0 ± 46.67 (NS)	122709.0 ± 6588.82 (NS)
Agonist AL-34662 (reference standard)	DMSO	50 nm*	136099.9 ± 2258.78*	NA	NA	NA
Agonist melanotan II acetate salt (reference standard)	DMSO	950 nm*	NA	11798.5 ± 27.58*	NA	NA
Agonist CP 55,940	DMSO	4.5 nm^1^	NA	NA	NA	144375.5 ± 7012.38
Agonist MCH	DMSO	62.5 nm^1^	NA	NA	545.0 ± 1.41	NA
Antagonist AM630 (reference standard)	DMSO	173.2 nm*	NA	NA	NA	4025.4 ± 994.31*
Antagonist GW-803430 (reference standard)	DMSO	18.1 nm*	NA	NA	1294.5 ± 142.13*	NA

RLU*: *relative luminescence units; NA: not applicable; *indicates a concentration at which maximum response of reference controls was observed; ^1^indicates EC_80_ concentration of agonist against which *N.  nucifera* petal extracts/reference controls were evaluated for their antagonistic potential.

*P* < 0.05.

NS: not significant.
